# *In Vivo* and *in Vitro* Effects of Secondary Metabolites against *Xanthomonas campestris* pv. *campestris*

**DOI:** 10.3390/molecules180911131

**Published:** 2013-09-11

**Authors:** Pablo Velasco, Margarita Lema, Marta Francisco, Pilar Soengas, María Elena Cartea

**Affiliations:** 1 Genetics, Breeding and Biochemistry of Brassicas, Misión Biológica de Galicia (MBG-CSIC), P.O. Box 28, E-36080 Pontevedra, Spain; 2 Department of Cellular Biology and Ecology, University of Santiago de Compostela, Praza do Obradoiro s/n, E-15782 Santiago de Compostela, Spain

**Keywords:** black rot, turnip greens, induced resistance, secondary metabolites, glucosinolates, phenolics

## Abstract

*Brassica rapa* is a crucifer that is grown worldwide, mainly as a vegetable. The quality of *B. rapa* crops is highly affected by the disease caused by the bacteria *Xanthomonas campestris* pv. c*ampestris* (Xcc). Glucosinolates and phenolic compounds can confer resistance to *Brassica* crops against pests and diseases, but few works have been done to evaluate their role in Xcc resistance. The objectives of this work were: (1) to evaluate the *in vivo* and *in vitro* antibacterial effect of gluconapin, its isothiocyanate and the methanolic extracts of *B. rapa* against the type 4 of Xcc, and (2) to test if there is induced resistance mediated by glucosinolates or phenolic compounds in two varieties of *B. rapa.* Gluconapin and its ITC varieties had an antibacterial effect on the development of Xanthomonas and this effect was strongly dependent on the concentration applied. Methanolic extracts from *B. rapa*, containing glucosinolates and phenolic compounds, inhibited the growth of these bacteria. Concentration of gluconapin is higher in resistant plants than in the susceptible ones and there is an induction of gluconapin, some flavonoids and sinapic acid 48 to 72 h after inoculation. Gluconapin plays a role in the constitutive resistance to Xcc, while gluconapin, some flavonoids and hydroxycinnamic acids are induced by a Xcc infection but it is not clear if this induction confers resistance to this disease.

## 1. Introduction

*Brassica rapa* L. is a crucifer that is grown worldwide, mainly as a vegetable and for edible and industrial oil, providing a large proportion of the daily food intake in many regions of the World. Different crops are grown for their leaves (turnip greens, Chinese cabbage, pak-choi, Narinosa, Komatsuna, Mizuna, Mibuna), seeds (turnip rape, Chinese turnip rape, Sarson types), inflorescences (Broccoletto, Cima di rapa, Caixin), floral shoots and stems (turnip tops, Zicaitai), and enlarged roots (turnips). World vegetable brassica production in 2010 was around 86.2 million tons [1]. China is the major *Brassica* producer in the World, accounting for more than 41.1 million t in 2010. This country, together with other Asian countries, cultivates mainly *B. rapa* crops, which reflects the importance of this vegetable [2,3].

However, the productivity and the quality of these crops are highly affected by several diseases, which result in substantial economic losses. Black rot, caused by the bacteria *Xanthomonas campestris* pv. *campestris* (Pammel) Dowson (Xcc), is one of the most devastating diseases in *Brassica* crops worldwide. The seedborne pathogen can survive in crop debris or crucifer weeds and it is especially damaging in vegetable *Brassica* crops like *B. rapa*, mainly in warm and humid climates [4]. The disease debilitates the plant, thus favoring the attack of other pathogens but even in mild attack, can cause several V-shaped necrotic lesions on leaves, which decrease the quality of the product for fresh market.

There are nine types of this species, being types 1 to 6 described by Vicente *et al.* [5] and 7 to 9 by Fargier and Manceau [6]. It is recognized that types 1 and 4 are the most widespread, accounting for the most part of the black rot disease around the world [5]. In NW Spain, black rot has been recently identified in several *Brassica* crops [7] including *B. rapa* subsp. *rapa*, being type 4 the most frequent in this environment. Recently, Lema et al. [8] evaluated a collection of *B. rapa* subsp. *rapa* accessionss currently kept at ‘Misión Biológica de Galicia’ (MBG-CSIC, Spain), for resistance to Xcc, races 1 and 4. They found some accessions with high levels of resistance to type 4 and partial resistance to type 1. The next step in the research would be to study the causes of the plant resistance to this disease.

Glucosinolates are nitrogen- and sulphur-containing plant secondary metabolites that occur mainly in the *Brassicaceae* family. Glucosinolates are β-thioglucoside *N*-hydroxysulphates containing a side chain and a β-d-glucopyranosyl moiety. Upon cellular disruption, glucosinolates are hydrolyzed to various bioactive breakdown products by the endogenous enzyme myrosinase. Isothiocyanates and indole glucosinolate metabolites (in particular indol-3-carbinol) are two major groups of autolytic breakdown products of glucosinolates. It is believed that glucosinolates can confer resistance to *Brassica* crops against pests and diseases [9,10,11,12]. Bending and Lincoln [13] also demonstrated the toxic properties of crucifer tissues after their incorporation into soil, thus limiting the growth of weeds, fungus and nematodes. Few *in vitro* and *in vivo* studies have been conducted to evaluate the effect of glucosinolates and/or their hydrolysis products on different *Brassica* diseases. Giamoustaris and Mithen [14] tested the hypothesis that *Brassica napus* L., with high levels of glucosinolates, would be more resistant to *Alternaria* spp. and *Leptosphaeria maculans* than varieties with low levels of them*.* Nevertheless, they found that *Alternaria* was favored by a high level of glucosinolates.

Due to the biocide effect of glucosinolates, different authors have tested the effect of isothiocyanates (ITCs) and glucosinolates on soil pathogens, by incorporating *Brassica* residues into soil or by testing their effect by using *in vitro* assays. ITCs have a positive effect in reducing soil pathogens, but a different persistence depending on the compound [14,15,16]. Brader *et al.* [17] reported that the accumulation of glucosinolates in *Arabidopsis thaliana* L. enhances resistance to *Erwinia carotovora* (Jones) and *Pseudomonas syringae* pv. *maculicola* (McCulloch). Recently, Aires *et al.* [18] evaluated the *in vitro* effect of glucosinolate hydrolysis products (GHP) on six plant pathogenic bacteria (*Agrobacterium tumefaciens*, *Erwinia chrysanthemi*, *Pseudomonas cichorii*, *Pseudomonas tomato*, *X. campestris* and *Xanthomonas juglandis*), showing that GHP could be an alternative tool in controlling these plant diseases. Besides, different authors have shown that secondary metabolites can act not only as a constitutive defense, but also as an induced defense. In this sense, glucosinolates have been indicated to be induced by the attack of herbivore species [19,20], but, as far as we know, no research has been done to evaluate the effect of glucosinolates and ITCs on the induced resistance against pathogens.

Besides glucosinolates, plant phenolics have been suggested to be associated to resistance against different plant diseases, but few works have been done in *Brassica* species. Hafidh *et al.* [21] evaluated the effect of methanolic crude extract of *B. oleracea* against 22 human pathogenic bacteria and fungi and they concluded that the extract showed antibacterial and antifungal activity. Other authors studied the effect of total phenolics on the growth of *X. campestris* [22] but no correlation was found.

The objectives of this work were: (1) to evaluate the *in vitro* antibacterial effect of gluconapin (GNA), which is the main glucosinolate in *B. rapa*, its glucosinolate enzymatic hydrolysis product, and a methanolic extract of *B. rapa* against *X. campestris* pv. *campestris* (Xcc), and (2) to test if there is induced resistance mediated by glucosinolates or phenolic compounds in *B. rapa*.

## 2. Results

### 2.1. *In Vitro* Effect of Gluconapin and Its Isothiocyanate

**Figure 1:**
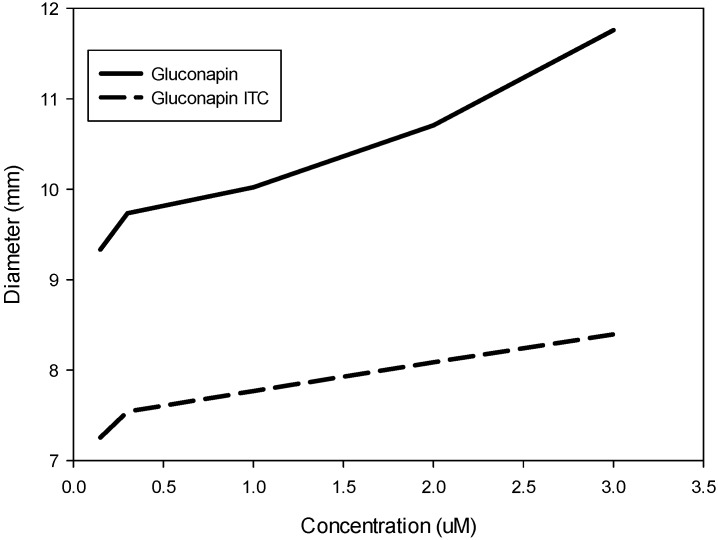
Effect of gluconapin and its isothiocyanate on the growth of *Xanthomonas campestris* pv. *campestris*, measured as the diameter (mm) of the inhibition zone circle.

There were significant differences for the inhibition zone diameter among concentrations and between the two compounds tested, GNA and GNA-ITC (*p* ≤ 0.001), but there was no significant concentration × compound interaction (*p* ≤ 0.1347). The inhibitory effect of gentamicin (15.54 mm) was significantly higher than those observed for the highest concentrations of gluconapin and its ITC (11.76 and 8.40 mm, respectively). The mean inhibition diameter of all concentrations for GNA was significantly higher than that obtained for its ITC (10.25 mm and 7.78 mm, respectively). The inhibition diameter of bacterial growth for both compounds through different concentrations is shown in Figure 1. From these results, we have demonstrated that gluconapin and its ITC had an antibacterial effect on the development of Xcc and that this effect was strongly dependent on the concentration. Differences on the bacterial growth inhibition observed between both compounds could be due to two main reasons. Firstly, isothiocyanates are unstable and volatile, so it is possible that the effect is lower than the effect of the glucosinolate. Secondly, the degradation of glucosinolates can render different compounds other than ITCs, which have not been tested and may have a stronger inhibitory effect.

### 2.2. *In Vitro* Effect of Glucosinolates and Phenolic Compounds in Methanolic Extracts from Leaves

**Figure 2:**
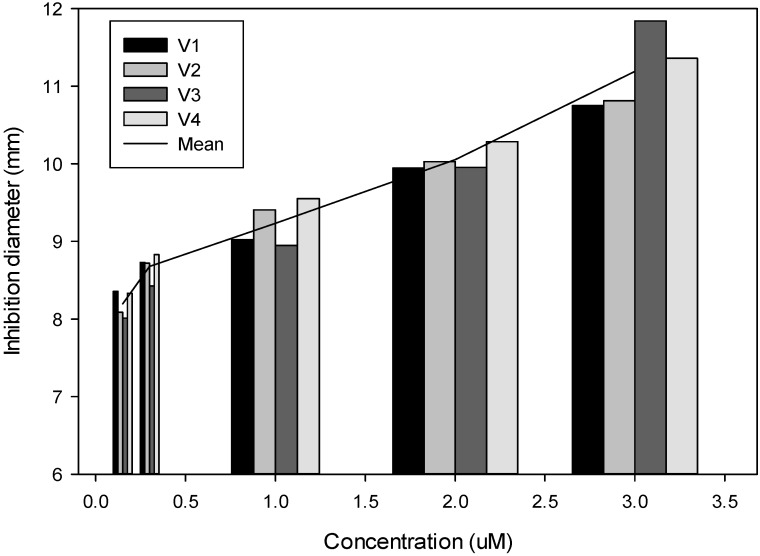
Effect of the methanolic extraction of four *Brassica rapa* varieties on the growth of *Xanthomonas campestris* pv. *campestris* measured as the diameter (mm) of the inhibition circle.

This experiment was designed to check if methanolic extracts from *B. rapa* leaves, which include glucosinolates and phenolic compounds, are effective against Xcc development. An analysis of variance was made to compare the effect of five different concentrations with regard to the control gentamicin. The mean highest concentration produced a mean inhibition diameter of 11.11 mm, which was significantly lower than that of gentamicin (15.36 mm). The effect of the extracts was dose-dependent, varying the inhibition diameter from 8.01 mm with the lowest concentration to 11.84 mm with the highest concentration (Figure 2).

### 2.3. Effect of Plant Glucosinolates and Phenolic Compounds

**Figure 3:**
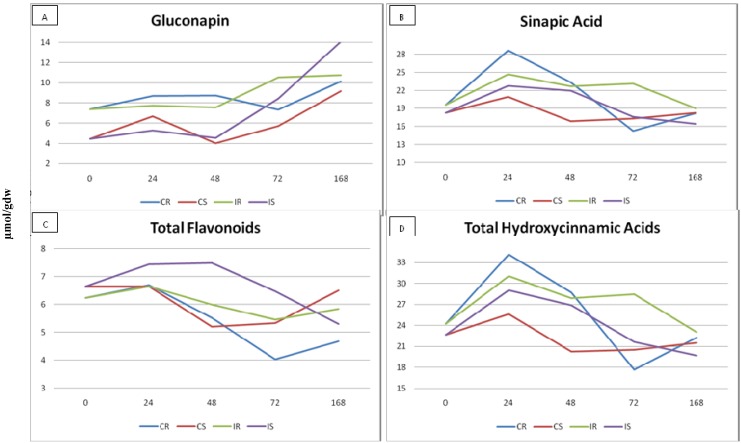
Content (µmol/g dw) of gluconapin (3A), sinapic acid (3B), total flavonoids (3C) and total hydroxycinnamic acids (3D) in control (C) and inoculated (I) plants of two *Brassica rapa* varieties, one resistant (R) and one susceptible (S) to race 4 of *Xanthomonas campestris* pv. *campestris*.

For this experiment, two *B. rapa* varieties were selected, being one resistant (MBG-BRS0259) and one susceptible (MBG-BRS0197) to Xcc race 4. [8]. Aproximately 100 plants of each variety were inoculated with Xcc and 100 plants of each variety were kept as control (without inoculation). All of them were evaluated for phenolic and glucosinolate composition five different times (five bulks and three replicates inside the bulk). The variation in time of the main compounds for resistant (R) and susceptible (S) varieties, inoculated (I) and non-inoculated (C), is shown in Figure 3. Twenty-one compounds (eight glucosinolates, six flavonoids and seven hydroxycinnamic acids) were identified by retention time and spectra and quantified (Table 1), based on a previous identification made in our laboratory by Francisco *et al.* [23]. At time 0, before inoculation with Xcc, there were significant differences between the resistant and the susceptible varieties for GNA (Table 1), which is the main glucosinolate in *B. rapa*. The resistant variety showed a higher concentration for this glucosinolate (7.39 µm/g dw) than the susceptible one (4.46 µm/g dw). This result could suggest a constitutive resistance to Xcc in *B. rapa* mediated by this glucosinolate.

After inoculation, glucosinolates were measured four different times in inoculated and non-inoculated plants on each variety. At time 1 (24 h), there were significant differences between the two varieties for GNA. Besides, differences were detected for progoitrin, the flavonoid kaempferol-3-*O*-(metoxycaffeoyl)sophoroside-7-*O*-glucoside and the hydroxycinnamic acid AC1 (a chlorogenic acid derivative). Regarding to phenolic compounds, in the comparison of means for AC1, inoculated plants showed a higher concentration than controls, thus suggesting an induced effect by Xcc.

**Table 1:** Analysis of variance of individual glucosinolates and phenolics identified in two varieties of *Brassica rapa*, MBG-BRS0259 (resistant to Xcc type 4) and MBG-BRS0155 (susceptible) in two different conditions, non-inoculated (time 0) and inoculated with Xcc (type 4) four different times after inoculation.

Compound	Time 0 (0 h)	Time 1 (24 h)	Time 2 (48 h)	Time 3 (72 h)	Time 4 (168 h)
GIB	0.060 **	0.040	0.435	0.005	0.334
PRO	0.004	1.884 *	1.168 **	0.145	0.168
GNA	12.877 **	6.329 *	15.742 *	5.737	8.894
OHGBS	0.045	0.034	0.021	0.012	0.011
GBN	0.004	0.001	0.011	0.010	0.030
GBS	0.000	0.036	0.029	0.003	0.011 *
GNT	0.001	0.005	0.007	0.001	0.011 *
NEOGBS	0.000	0.000	0.000 *	0.001	0.000
TGLS	17.579 **	13.987 **	20.074 *	5.912	6.037
CQAC	0.000	0.001	0.008	0.003	0.005
PCOQAC	0.000	0.000	0.028 **	0.004	0.021 **
F1	0.062	0.066 *	0.246 **	0.132	0.042
F2	0.104	0.183	0.640 **	0.252	0.135 *
F3	0.001	0.006	0.013	0.039	0.050 **
F4	0.020	0.015	0.026	0.058	0.057 *
F5	0.001	0.003	0.020 *	0.009 **	0.008
F6	0.000	0.003	0.008 *	0.003	0.002
AC1	0.000	0.528 **	0.405 **	0.063	0.033
AC2	0.135	0.646	1.229	0.793	0.304
SA	2.509	33.127	25.903 *	13.874	1.955
S1	0.000	0.007	0.104	0.038	0.021
S2	0.000	0.009	0.174 **	0.029	0.021
TFLAV	0.248	0.459	3.053 **	1.981	1.178
THYDROX	3.745	38.488	44.937 *	26.346	3.362
TPHEN	2.089	37.815	55.227 *	35.745	4.537

* Significant at *p* ≤ 0.05, ** Significant at *p* ≤ 0.01. GIB = Glucoiberin; PRO = Progoitrin; GNA = Gluconapin; OHGBS = 4-hydroxyglucobrassicin; GBN = Glucobrassicanapin; GBS = Glucobrassicin; GNT = Gluconasturtiin; NEOGBS = Neoglucobrassición; TGLS = Total glucosinolates; CQAC = 3-caffeoyl-quinnic acid; PCOQAC = 3-paracoumaroyl-quinnic acid; F1 = Kaempferol-3-*O*-(metoxycaffeoyl)sophoroside-7-*O*-glucoside; F2 = Kaempferol-3-*O*-(caffeoyl)sophoroside-7-*O*-glucoside; F3 = Kaempferol-3-*O*-(sinapoyl)sophoroside-7-*O*-glucoside; F4 = Kaempferol-3-*O*-(feruloyl)sophoroside-7-*O*-glucoside; F5 = Kaempferol-3-*O*-(*p*-coumaroyl)sophoroside-7-*O*-glucoside; F6 = Kaempferol-3,7-di-*O*-glucoside; AC1 = Chlorogenic acid derivative; AC2 = Sinapic acid derivative; SA = Sinapic acid; S1 = 1,2-disinapoylgentiobioside; S2 = 1-sinapoyl-2-feruloylgentiobioside; TFLAV = Total flavonoids; THYDROX = Total hydroxycinnamic acids, TPHEN = Total phenolics.

After 48 h (time 2), differences among varieties were significant for 15 compounds (Table 1). Regarding glucosinolates, we could observe that resistant plants (I and C) had more GNA than susceptible ones. An increase in GNA concentration was observed in inoculated plants from 48 to 72 h, thus suggesting a role of GNA in the resistance to Xcc (Figure 3A). At this time, concentration of GNA is higher in the inoculated plants than in the control plants. With regard to flavonoids, there were not significant differences among non-inoculated resistant and susceptible plants. When inoculated, resistant plants did not increase the flavonoid concentration but susceptible plants increased the concentration in 43%, showing that there is an induction of these compounds after inoculation with Xcc (Figure 3C). For hydroxycinnamic acids there were significant differences among non-inoculated resistant and susceptible plants, which indicate a possible role of hydroxycinnamic acids in the constitutive resistance to Xcc. This effect was due to the sinapic acid, which has a higher concentration in the resistant plants (23.3 um/g dw) compared to the susceptible plants (16.88 um/g dw) (Figure 3B). Like the case of flavonoids, 48 hours after inoculation, resistant plants did not increase their content, but susceptible plants increased their content in 33% (Table 2).

**Table 2:** Means for glucosinolate and phenolic content (µmol/g dw) in two varieties of *Brassica rapa*, MBG-BRS0259 (resistant) and MBG-BRS0155 (susceptible) in two different conditions non-inoculated (control) and inoculated with Xcc race 4, 48 h after inoculation.

Compound	Resistant Inoculated	Resistant Control	Susceptible Inoculated	Susceptible Control	LSD
PRO	0.000b	0.793a	0.000b	1.263a	0.650
GNA	7.567ab	8.730a	4.563bc	4.010c	3.389
NEOGBS	1.077a	1.060c	1.070ab	1.053c	0.012
TGLS	15.170ab	17.203a	11.480b	12.540b	4.086
PCOQAC	0.067b	0.203a	0.000b	0.000b	0.111
F1	1.113b	0.890b	1.493a	0.883b	0.234
F2	1.743b	1.430b	2.477a	1.430b	0.393
F5	0.537b	0.463b	0.650a	0.493b	0.108
F6	0.713a	0.677a	0.703a	0.597b	0.074
AC1	1.533a	1.097b	1.780a	1.000b	0.378
SA	22.673a	23.330a	21.930a	16.880b	4.531
S2	0.150bc	0.553a	0.000c	0.350ab	0.280
TFLAV	5.990b	5.528b	7.487a	5.207b	0.948
THYDROX	27.890a	28.717a	26.907a	20.240b	5.706
TPHEN	33.877a	34.247a	34.390a	25.447b	6.236

In each row, means followed by the same letter are not significantly different at *p* ≤ 0.05. PRO = Progoitrin; GNA = Gluconapin; NEOGBS = Neoglucobrassición; TGLS = Total glucosinolates; PCOQAC = 3-paracoumaroyl-quinnic acid; F1 = Kaempferol-3-*O*-(metoxycaffeoyl)sophoroside-7-O-glucoside; F2 = Kaempferol-3-*O*-(caffeoyl)sophoroside-7-*O*-glucoside; F5 = Kaempferol-3-*O*-(p-coumaroyl)sophoroside-7-*O*-glucoside; F6 = Kaempferol-3,7-di-*O*-glucoside; AC1 = Chlorogenic acid derivative; SA = Sinapic acid; S2 = 1-sinapoyl-2-feruloylgentiobioside; TFLAV = Total flavonoids; THYDROX = Total hydroxycinnamic acids; TPHEN = Total phenolics.

## 3. Discussion

Over the last few years, several authors have evaluated the effects of glucosinolates or glucosinolate hydrolysis products (GHP) on different plant diseases, because of their control activity against several plant pathogens, insects and nematodes in *Brassica* crops [16,24,25,26,27,28,29,30] or *Arabidopsis* [31]. Most studies are inconclusive but in many of them it is possible to observe an inhibitory effect of glucosinolates, varying with compounds and diseases.

Little is known about the causes of resistance to Xcc in brassica crops. In our study, we hypothesized that glucosinolates and phenolic compounds may have a role in resistance to this pathogen. Recently, Jiang *et al.* [32] constructed a cDNA library by using a resistant line and its susceptible near-isogenic ones to study sequences related to resistance to Xcc. They found at least 12 genes with different expression in resistant and susceptible lines, but no gene or sequence appears to be related with glucosinolate synthesis. Nevertheless, different authors have evaluated the role of glucosinolates in the defense against Xcc. Aires *et al.* [18] evaluated the effect of different glucosinolate hydrolysis products (GHPs) against several phytopathogenic bacteria, including Xcc. They found a strong effect of GHPs, meaning that the growth of Xcc could be limited by the addition of GHPs, especially allyl ITC, benzyl ITC, sulforaphane and indol-3-carbinol. Contrary to our results, they also found that some ITCs had a stronger effect than gentamicin. Our results confirm that pure GNA and its ITC interfere in the development of Xcc. The effect was dose-dependent and lower than the antibiotic employed as control.

As it was shown in the experiment, methanolic extracts containing glucosinolates and phenolics had an inhibitory effect on the development of Xcc. Differences among varieties extracts were not significant but there was a significant effect of the concentration, following a linear tendency.

Once we have demonstrated that pure GLS and plant extracts enriched in GLS and phenolics have a role in the inhibition of Xcc growth, it would be convenient to investigate the role of these compounds in the plant defense against this pathogen. From our results, we conclude that glucosinolates, mainly GNA, have a role in the resistance to Xcc and that a strong induction occurs from 48 to 72 h. Regarding phenolics, only sinapic acid may have a role in constitutive defense, but most of other phenolic compounds are induced in susceptible plants after inoculation. The accurate function of this induction is still unknown. Recently, Aires *et al.* [22] studied the potential role of glucosinolates and their respective hydrolysis products against Xcc infection. They evaluated the variation in profile and content of GLS and total phenolics before and after inoculation with Xcc to evaluate how the initial and post-inoculation profiles correlate with the development of Xcc. Their results conclude that aliphatic and indole GLS may play a complex role in the defense depending on the plant species. In this study, no relationship was found between total polyphenolic content and resistance to Xcc. Other authors studied the metabolite induction by *Verticillium dahliae* in broccoli and cauliflower [33]. They could not observe glucosinolate induction for either broccoli or cauliflower but they found induction mediated by phenolic and lignin in broccoli. Inoculated plants showed a higher content of phenolic and lignin than non-inoculated plants. This was related with the increased resistance of broccoli, being an example of induced resistance.

## 4. Experimental

### 4.1. *In Vitro* Effect of GNA and Its Isothiocyanate

Pure extracts of GNA (Phytoplan Diehm & und Neuberger GmbH, Heidelberg, Germany) and its isothiocyanate (GNA-ITC, TCI Europe, Zwijndrecth, Belgium) in five different doses (0.15, 0.30, 1.0, 2.0 and 3.0 μL mL^−1^) of each product were tested [18]. Bacterial isolates of type 4 strain HRI1279A (collected from *B. oleracea* var. *capitata* in the UK) typed by Vicente *et al.* [5] and provided by WHRI-Wellesbourne, UK, were used. Xcc conserved in glycerol (20%) at −80 °C was picked and plated in Petri dishes containing 20 mL of fresh Potato Dextrose Agar (PDA). After incubation for 24 h at 30 °C, Xcc were subcultured in nutrient broth (NB) at 30 °C in darkness and with agitation. After overnight incubation, 200 μL of suspension were spread uniformly in 9 cm Petri dishes containing PDA medium by using a sterile plastic inoculation loop. Six sterile filter paper discs (6 mm in diameter, Oxoid, Hampshire, UK) impregnated with 15 μL of each compound tested at each dose and a positive control (10 µg disc^−1^ of commercial gentamicin) were placed on each plate by using a disc dispenser (Oxoid). A disc containing the negative control (15 μL of solvent dimethyl sulfoxide, DMSO) was placed in the center of each plate. After incubation for18 h at 30 °C, the inhibition of Xcc growth was measured as the diameter (in mm) of the zone without bacteria around the disc, by using a digital caliper (Metrica, Barcelona, Spain). For each compound, five replications were made and the antibacterial activity assessment was expressed as the mean of inhibition zone diameters (mm) according to Aires *et al.* [18].

### 4.2. *In Vitro* Effect of Glucosinolates and Phenolic Compounds in Methanolic Extract from Leaves

Methanolic extracts from *B. rapa* leaves, which contain glucosinolates and phenolic compounds as major phytochemicals, were tested against Xcc to check their possible antibacterial effect against this disease and to compare them to the effect caused by pure extracts of GNA and its isothiocyanate (GNA-ITC). Four *B. rapa* varieties, representative of the *B. rapa* collection from NW Spain, were tested in five different concentrations (1 mL of the methanolic extraction explained below was diluted by 3, 10, 100, 1000 and 10,000). These varieties are kept at the germplasm collection at MBG-CSIC and are commonly distributed between farmers because of their good agronomic performance as turnip tops or turnip greens [34]: MBG-BRS0082, 0143, 0472, 0550. An experiment was performed and the antibacterial activity was assessed as it was previously explained in the *in vitro* assay of GNA.

### 4.3. Effect of Plant Glucosinolates and Phenolic Compounds

Two *B. rapa* varieties from the *Brassica* collection maintained at the MBG-CSIC were selected, being one resistant (MBG-BRS0259) and one susceptible (MBG-BRS0155) to Xcc type 4 [8]. Varieties were inoculated with Xcc isolate HRI1279A (corresponding to type 4 type strain) under greenhouse conditions. For all experiments, inoculum was prepared by growing the bacterial isolate HRI1279A onto screening 523 medium plates for 48 h at 30 °C and preparing a cell suspension in sterile tap water with a concentration of 5 × 10^8^ CFU mL^−1^. This concentration was measured in a Beckman Coulter DU 6 spectrophotometer and corresponded with an optical density of 0.50 at 600 nm.

Hundred plants per genotype were inoculated 5 weeks after planting by using the multiple needles method according to Lema-Marquez *et al.* [35]. After inoculating the youngest leaf of each plant, greenhouse conditions were maintained at 14 h of light, mean temperature 24 °C at night and 28 °C during the day and relative humidity between 80% and 90%. In order to study the possible role of glucosinolates and phenolic compounds on plant resistance and, moreover, to determine if this resistance is constitutive or induced, glucosinolate and phenolic concentrations were measured five different times: before the inoculation (time 0) and after the inoculation (time 1 = 24 h, time 2 = 48 h, time 3 = 72 h and time 4 = 168 h) in leaves of a set of inoculated plants and in a set of non-inoculated plants. Then, for statistical analysis the experiment is composed of four different treatments: resistant variety MBG-BRS0259 inoculated (RI), non-inoculated (RC), susceptible variety MBG-BRS0155 inoculated (SI) and non-inoculated (SC).

### 4.4. Extraction and Determination of Glucosinolates and Phenolic Compounds

The LC gradient for glucosinolate and phenolic analyses is a multipurpose chromatographic method that simultaneously separates glucosinolates and phenolics, and it was recently applied to *Brassica* crops [23,36]. A portion of 150 mg of each sample was extracted in 70% MeOH (4 mL) at 70 °C for 30 min with vortex mixing every 5 min to facilitate the extraction. The samples were centrifuged (13,000g, 15 min), 1 mL of supernatants was collected, and methanol was completely removed by using a sample concentrator (DB-3D, Techne, Staffordshire, UK) at 70 °C. The dry material obtained was redissolved in 1 mL of ultrapure water and filtered through a 0.20 μm syringe filter (Acrodisc Syringe Filters, Pall Life Sciences, PortWashington, NY, USA). Chromatographic analyses were carried out on a Luna C18 column (250 mm × 4.6 mm, 5 μm particle size; Phenomenex, Macclesfield, UK). The mobile phase was a mixture of (A) ultrapure water/trifluoroacetic acid (TFA) (99.9:0.1) and (B) methanol/TFA (99.9:0.1). The flow rate was 1 mL min-1 in a linear gradient starting with 0% B after 0–5 min, reaching 17% B after 15–17 min, 25% B after 22 min, 35% B after 30 min, 50% B after 35 min, 99% B after 50 min, and 0% B after 55–65 min. The injection volume was 20 μL, and chromatograms were recorded at 330 nm for phenolic derivatives and at 227 nm for glucosinolates in a model 600 HPLC instrument (Waters Corporation, Millford, MA, USA) equipped with a model 486 UV tunable absorbance detector (Waters). Glucosinolates were quantified by using sinigrin (sinigrin monohydrate from Phytoplan, Diehm and Neuberger GmbH, Heidelberg, Germany) as standard. Caffeoylquinic and *p*-coumaroylquinic acid derivatives were quantified as chlorogenic acid (5-caffeoylquinic acid, Sigma-Aldrich Chemie GmbH, Steinheim, Germany), flavonoids as kaempferol 3-rutinoside (Extrasynthese, Genay, France), and sinapic acid and derivatives as sinapic acid (Sigma).

### 4.5. Statistical Analysis

For all experiments, analyses of variance and mean comparisons were made for the inhibition zone diameter. Mean values were separated by using Fisher’s protected least significant difference (LSD) at the 0.05 level of probability. Statistical analyses were performed by using the SAS statistical package [37].

## 5. Conclusions

Pure gluconapin, its ITC and methanolic extracts from *B. rapa*, containing glucosinolates and phenolic compounds, clearly inhibited the growing of Xcc, being this effect strongly dependent on the concentration applied. The concentration of gluconapin is higher in resistant plants than in the susceptible ones, but not for phenolic compounds, indicating that gluconapin plays a role in the constitutive resistance to Xcc. Gluconapin, some flavonoids and hydroxycinnamic acids are induced by Xcc infection but it is not clear if this induction confers resistance to this disease. Further studies about the metabolic effect of glucosinolates and phenolics would be necessary to understand the effect of these metabolites on the development of Xcc.

## References

[1] FAOSTAT.

[2] Kim J., Jung Y., Song B., Bong Y.-S., Ryu D.H., Lee K.-S., Hwang G.-S. (2013). Discrimination of cabbage (*Brassica rapa* ssp. *pekinensis*) cultivars grown in different geographical areas using ^1^H-NMR-based metabolomics. Food Chem..

[3] Bong Y.-S., Shin W.-J., Gautam M.K., Jeong Y.-J., Lee A.-R., Jang C.-S., Lim Y.-P., Chung G.-S., Lee K.-S. (2012). Determining the geographical origin of Chinese cabbages using multielement composition and strontium isotope ratio analyses. Food Chem..

[4] Dias J.S., Nogueira P., Corvo L. (2010). Evaluation of a core collection of Brassica rapa vegetables for resistance to *Xanthomonas campestris* pv. campestris. Afr. J. Agric. Res..

[5] Vicente J.G., Conway J., Roberts S.J., Taylor J.D. (2001). Identification and origin of *Xanthomonas campestris* pv. campestris races and related pathovars. Phytopathology.

[6] Fargier E., Manceau C. (2007). Pathogenicity assays restrict the species *Xanthomonas campestris* into three pathovars and reveal nine races within *X. campestris* pv. campestris. Plant Pathol..

[7] Lema M., Elena Cartea M., Sotelo T., Velasco P., Soengas P. (2012). Discrimination of *Xanthomonas campestris* pv. campestris races among strains from northwestern Spain by *Brassica* spp. genotypes and rep-PCR. Eur. J. Plant Pathol..

[8] Lema M., Cartea M.E., Francisco M., Velasco P., Soengas P. (2013). Screening for resistance to black rot in a Spanish collection of *Brassica rapa*. Plant Breed..

[9] Fahey J.W., Zalcmann A.T., Talalay P. (2001). The chemical diversity and distribution of glucosinolates and isothiocyanates among plants. Phytochemistry.

[10] Agrawal A.A., Kurashige N.S. (2003). A role for isothyociantes in plant resistance against the specialist herbivore Pieris rapae. J. Chem. Ecol..

[11] Rahmanpour S., Backhouse D., Nonhebel H.M. (2009). Induced tolerance of Sclerotinia sclerotiorum to isothiocyanates and toxic volatiles from *Brassica* species. Plant Pathol..

[12] Fan Z.X., Lei W.X., Sun X.L., Yu B., Wang Y.Z., Yang G.S. (2008). The association of *Sclerotinia sclerotiorum* resistance with glucosinolates in Brassica napus double-low DH population. J. Plant Pathol..

[13] Bending G.D., Lincoln S.D. (2000). Inhibition of soil nitrifying bacteria communities and their activities by glucosinolate hydrolysis products. Soil Biol. Biochem..

[14] Giamoustaris A., Mithen R. (1997). Glucosinolates and disease resistance in oilseed rape (*Brassica napus* ssp. *oleifera*). Plant Pathol..

[15] Lazzeri L., Manici L.M. (2001). Allelopathic effect of glucosinolate containing plant green manure on *Pythium* sp. and total fungal population in soil. HortScience.

[16] Motisi N., Montfort F., Dore T., Romillac N., Lucas P. (2009). Duration of control of two soilborne pathogens following incorporation of above- and below-ground residues of Brassica juncea into soil. Plant Pathol..

[17] Brader G., Mikkelsen M.D., Halkier B.A., Palva E.T. (2006). Altering glucosinolate profiles modulates disease resistance in plants. Plant J..

[18] Aires A., Mota V.R., Saavedra M.J., Monteiro A.A., Simoes M., Rosa E.A.S., Bennett R.N. (2009). Initial *in vitro* evaluations of the antibacterial activities of glucosinolate enzymatic hydrolysis products against plant pathogenic bacteria. J. Appl. Microbiol..

[19] Mathur V., Ganta S., Raaijmakers C.E., Reddy A.S., Vet L.E.M., van Dam N.M. (2011). Temporal dynamics of herbivore-induced responses in *Brassica juncea* and their effect on generalist and specialist herbivores. Entomol. Exp. Appl..

[20] Broekgaarden C., Voorrips R.E., Dicke M., Vosman B. (2011). Transcriptional responses of *Brassica nigra* to feeding by specialist insects of different feeding guilds. Insect Sci..

[21] Hafidh R.R., Abdulamir A.S., Vern L.S., Bakar F.A., Abas F., Jahanshiri F., Sekawi Z. (2011). Inhibition of growth of highly resistant bacterial and fungal pathogens by a natural product. Open Microbiol. J..

[22] Aires A., Dias C.S.P., Carvalho R., Oliveira M.H., Monteiro A.A., Simoes M.V., Rosa E.A.S., Bennett R.N., Saavedra M.J. (2011). Correlations between disease severity, glucosinolate profiles and total phenolics and *Xanthomonas campestris* pv. campestris inoculation of different Brassicaceae. Sci. Hortic..

[23] Francisco M., Moreno D.A., Cartea M.E., Ferreres F., Garcia-Viguera C., Velasco P. (2009). Simultaneous identification of glucosinolates and phenolic compounds in a representative collection of vegetable *Brassica rapa*. J. Chromatogr. A.

[24] O’Callaghan K.J., Stone P.J., Hu X.J., Griffiths D.W., Davey M.R., Cocking E.C. (2000). Effects of glucosinolates and flavonoids on colonization of the roots of Brassica napus by Azorhizobium caulinodans ORS571. Appl. Environ. Microbiol..

[25] Buskov S., Serra B., Rosa E., Sorensen H., Sorensen J.C. (2002). Effects of intact glucosinolates and products produced from glucosinolates in myrosinase-catalyzed hydrolysis on the potato cyst nematode (*Globodera rostochiensis* cv. Woll). J. Agric. Food Chem..

[26] Menard R., Larue J.P., Silue D., Thouvenot D. (1999). Glucosinolates in cauliflower as biochemical markers for resistance against downy mildew. Phytochemistry.

[27] Siemens J., Glawischnig E., Ludwig-Mueller J. (2008). Indole glucosinolates and camalexin do not influence the development of the clubroot disease in Arabidopsis thaliana. J. Phytopathol..

[28] Siemens J., Nagel M., Ludwig-Muller J., Sacristan M.D. (2002). The interaction of Plasmodiophora brassicae and Arabidopsis thaliana: Parameters for disease quantification and screening of mutant lines. J. Phytopathol..

[29] Kirkegaard J.A., Sarwar M., Wong P.T.W., Mead A., Howe G., Newell M. (2000). Field studies on the biofumigation of take-all by Brassica break crops. Aust. J. Agric. Res..

[30] Sarwar M., Kirkegaard J.A., Wong P.T.W., Desmarchelier J.M. (1998). Biofumigation potential of brassicas - III. *In vitro* toxicity of isothiocyanates to soil-borne fungal pathogens. Plant Soil.

[31] Tierens K., Thomma B.P.H., Brouwer M., Schmidt J., Kistner K., Porzel A., Mauch-Mani B., Cammue B.P.A., Broekaert W.F. (2001). Study of the role of antimicrobial glucosinolate-derived isothiocyanates in resistance of arabidopsis to microbial pathogens. Plant Physiol..

[32] Jiang H., Song W., Li A., Yang X., Sun D. (2011). Identification of genes differentially expressed in cauliflower associated with resistance to *Xanthomonas campestris* pv. campestris. Mol. Biol. Reports.

[33] Njoroge S.M.C., Vallad G.E., Park S.-Y., Kang S., Koike S.T., Bolda M., Burman P., Polonik W., Subbarao K.V. (2011). Phenological and Phytochemical Changes Correlate with Differential Interactions of Verticillium dahliae with Broccoli and Cauliflower. Phytopathology.

[34] Francisco M., Velasco P., Lema M., Cartea M.E. (2011). Genotypic and environmental effects on agronomic and nutritional value of Brassica rapa. Agron. J..

[35] Lema Marquez M., Teran H., Singh S.P. (2007). Selecting common bean with genes of different evolutionary origins for resistance to *Xanthomonas camplestris* pv. phaseoli. Crop Sci..

[36] Velasco P., Francisco M., Moreno D.A., Ferreres F., Garcia-Viguera C., Cartea M.E. (2011). Phytochemical Fingerprinting of Vegetable *Brassica oleracea* and *Brassica napus* by Simultaneous Identification of Glucosinolates and Phenolics. Phytochem. Anal..

[37] SAS (2008). SAS Online Doc, Version 9.2.

